# Detection of Coronary Artery Disease Using Multi-Domain Feature Fusion of Multi-Channel Heart Sound Signals

**DOI:** 10.3390/e23060642

**Published:** 2021-05-21

**Authors:** Tongtong Liu, Peng Li, Yuanyuan Liu, Huan Zhang, Yuanyang Li, Yu Jiao, Changchun Liu, Chandan Karmakar, Xiaohong Liang, Mengli Ren, Xinpei Wang

**Affiliations:** 1 School of Control Science and Engineering, Shandong University, Jinan 250061, China; tongtongliu@mail.sdu.edu.cn (T.L.); liuyy@sdu.edu.cn (Y.L.); zhanghuan@mail.sdu.edu.cn (H.Z.); 201814513@mail.sdu.edu.cn (Y.J.); changchunliu@sdu.edu.cn (C.L.); xiaohongliang0125@mail.sdu.edu.cn (X.L.); 201934551@mail.sdu.edu.cn (M.R.); 2 Division of Sleep and Circadian Disorders, Brigham and Women’s Hospital, Boston, MA 02115, USA; pli9@bwh.harvard.edu; 3 Division of Sleep Medicine, Harvard Medical School, Boston, MA 02115, USA; 4 School of Instrument Science and Engineering, Southeast University, Nanjing 210096, China; 230209207@seu.edu.cn; 5 Department of Medical Engineering, Shandong Provincial Hospital Affiliated to Shandong First Medical University, Jinan 250021, China; 6 School of Information Technology, Deakin University, Geelong, VIC 3225, Australia; karmakar@deakin.edu.au

**Keywords:** heart sound, coronary artery disease, multi-channel, entropy, cross entropy

## Abstract

Heart sound signals reflect valuable information about heart condition. Previous studies have suggested that the information contained in single-channel heart sound signals can be used to detect coronary artery disease (CAD). But accuracy based on single-channel heart sound signal is not satisfactory. This paper proposed a method based on multi-domain feature fusion of multi-channel heart sound signals, in which entropy features and cross entropy features are also included. A total of 36 subjects enrolled in the data collection, including 21 CAD patients and 15 non-CAD subjects. For each subject, five-channel heart sound signals were recorded synchronously for 5 min. After data segmentation and quality evaluation, 553 samples were left in the CAD group and 438 samples in the non-CAD group. The time-domain, frequency-domain, entropy, and cross entropy features were extracted. After feature selection, the optimal feature set was fed into the support vector machine for classification. The results showed that from single-channel to multi-channel, the classification accuracy has increased from 78.75% to 86.70%. After adding entropy features and cross entropy features, the classification accuracy continued to increase to 90.92%. The study indicated that the method based on multi-domain feature fusion of multi-channel heart sound signals could provide more information for CAD detection, and entropy features and cross entropy features played an important role in it.

## 1. Introduction

Coronary artery disease (CAD) has been the leading cause of death in cardiovascular disease globally [1] and is still increasing at an alarming rate. Therefore, there is an urgency to develop convenient and accurate options for CAD detection of large-scale populations. Coronary angiography (CAG) [2] is widely regarded as the gold standard for detecting CAD. But it is not suitable as a routine examination method for early screening due to its invasive and high price defects. Medical research has confirmed that when blood flows through the stenosis of a blood vessel, it will impact the wall of the blood vessel and form turbulence. The turbulence can cause murmurs in heart sound signals [3]. Therefore, as a non-invasive detection method, heart sound analysis has the potential to become a cost-effective screening tool to achieve the early detection of CAD [4].

The correlation between diastolic murmur and stenosis was proved by Akay et al. [5]. In that research, four analysis methods were used, including fast Fourier transform, auto-regressive, autoregressive moving average, and minimum norm. The results obtained by Semmlow et al. [6] also indicated that an above-normal percentage of high-frequency en-ergy is closely related to narrowed coronary arteries. Some researchers used heart sound-based risk assessment to help detect CAD, and the results demonstrated the poten-tial use of heart sound to identify CAD [7,8]. In order to make further use of diastolic murmurs for CAD detection, many scholars analyzed diastolic heart sound signals in the frequency domain. Schmidt et al. [9] analyzed the frequency distribution of the diastolic period, and identified new features to describe an increase in low-frequency power in CAD patients. Gauthier et al. [10] used the energy ratio of high and low-frequency com-ponents as classification features. Zhao et al. [11] proposed a novel approach based on Hilbert–Huang transform to analyze the diastolic murmurs of CAD. In addition to fre-quency-domain features, wavelet-based feature sets in the time-frequency domain are also used to classify abnormal heart sounds [12]. Nonlinear analysis is an effective way to re-flect nonlinearity and complexity. It was proven that the correlation dimension can be used for CAD detection [13]. As a nonlinear feature, entropy is very suitable for the analy-sis of non-stationary signals. Akay et al. [14] used approximate entropy of heart sounds to identify CAD. Among these studies based on single-channel heart sound signals, the highest accuracy of detecting CAD is 78%. The accuracy of detecting CAD based on heart sound signals needs to be further improved.

Considering multiple auscultation areas, researchers have collected heart sound sig-nals from multiple locations on the chest for disease detection [15]. Akanksha et al. [16] used a cross power spectrum to analyze the heart sound signals collected from four posi-tions of the chest for CAD detection. Rujoie et al. [17] diagnosed and assessed the severity of tricuspid regurgitation using heart sounds recorded in seven channels. Pathak et al. [18] collected multi-channel heart sounds and eliminated environmental noise by using the delayed propagation of heart sounds between different channels. Griffel et al. [19] evalu-ated the effect of automutual information function for CAD detection by using two-channel heart sound signals at three positions measured in sequence. The above studies confirmed that the results of the joint analysis of multi-channel heart sound sig-nals are better than those of a single channel.

The analysis of entropy measures can provide a valuable tool for quantifying the regularity of physiological time series [20]. Sample entropy (SampEn) and fuzzy entropy (FuzzyEn) are widely used in physiological signals for they overcome the shortcomings of ApEn, such as bias and relative inconsistency [21,22]. However, SampEn and FuzzyEn need to manually set parameters according to the data, which depends on experience and is not conducive to the standardization of the formula. Distribution entropy (DistEn) can preclude the dependence upon input parameters, and it has shown superiority for the analysis of short-term physiological signals compared to SampEn and FuzzyEn [23,24]. Cross entropy analysis can enable the measurement of the synchrony or similarity of patterns between two channel signals. Previous studies have shown that cross-sample entropy (XSampEn) [25], cross fuzzy entropy (FuzzyEn) [26], and joint distribution entropy (JDistEn) [27] have great potential for physiological signal analysis. These three cross entropy features have been developed from the above three entropy features, respectively, so these six features are explored in this study.

**Figure 1:**
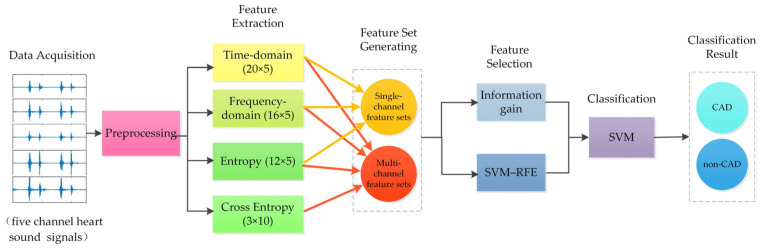
Block diagram of multi-domain feature fusion of multi-channel heart sound signals to detect CAD.

To improve the accuracy of detecting CAD based on heart sound signals, this paper collected five-channel heart sound signals, and then proposed a method based on multi-domain feature fusion of multi-channel heart sound signals to detect CAD. First, the time-domain, frequency-domain, entropy, and cross entropy features of heart sound signals were extracted as features, and different feature sets were composed of these features. Then, recursive feature elimination based on support vector machine (SVM–RFE) was used for feature selection, as it iteratively obtains the optimal feature subset. Meanwhile, information gain was also used for feature ranking. Subsequently, support vector machines (SVM) were used for classification. Results showed that this study provided an effective computer-aided method for the identification of CAD patients. Multi-channel feature, entropy feature, and cross entropy feature were all helpful for classification performance. Figure 1 depicts a system block diagram for detecting CAD using multi-domain feature fusion of multi-channel heart sound signals.

## 2. Materials and Methods

### 2.1. Data Acquisition

This study was conducted under the principles of the Helsinki Declaration and its subsequent amendments and obtained the approval of the Institutional Review Board (No. 034). All the subjects were from Qi Lu Hospital of Shandong University, and were provided with informed consent before participation. The inclusion criterion was subjects that were scheduled to undergo a CAG within two days. Three types of subjects were excluded from the study: (a) subjects who had previously undergone percutaneous coronary intervention or coronary artery bypass surgery, (b) subjects who had valvular heart disease verified by echocardiography, (c) subjects who had acute myocardial infarction. Subjects with ≥50% stenosis in at least one of three main coronary artery branches (i.e., left anterior descending, left circumflex, and right coronary artery) were categorized as CAD, otherwise as non-CAD. This study enrolled 36 subjects, including 21 CAD patients and 15 non-CAD subjects. All CAD patients had left anterior descending stenosis, in which there were 7 subjects with first diagonal branch stenosis, 2 subjects with second diagonal branch stenosis, 1 subject with septal artery stenosis, 1 subject with middle branch stenosis, and 16 subjects with left circumflex artery stenosis. The basic characteristics of all subjects are given in Table 1. Mann–Whitney U tests were used for continuous variables, for they did not conform to the normal distribution. Since the gender group is a binary categorical variable, and considering the sample size, this study adopted Fisher’s exact test as the statistical test method, and the *p* value is listed in Table 1.

A cardiovascular function detection device (CVFD, Huiyironggong Technology Co., Ltd., Jinan, China) was used to record the heart sound signals. Since CAD mostly occurs in the left coronary artery, under the recommendations of the guidelines and cardiovascular experts, an acquisition channel on the left was added on the basis of the original four auscultation areas. The five detectors of electronic stethoscope were respectively placed in the second intercostal space on the right edge of the sternum, the second intercostal space on the left edge of the sternum, the third intercostal space on the left edge of the sternum, the fourth intercostal space on the left edge of the sternum, and the intersection of the fourth intercostal space and the midclavicular line. For each subject, heart sound signals in five different locations were simultaneously recorded for 5 min at a sampling rate of 2 kHz. The collected data were numbered as channel 1 to channel 5. 

### 2.2. Signal Preprocessing

**Figure 2:**
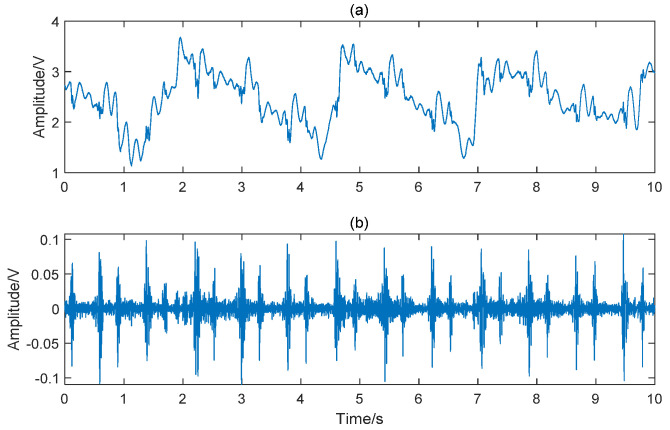
Comparison before and after preprocessing. (**a**) original signal; (**b**) preprocessed signal.

**Figure 3:**
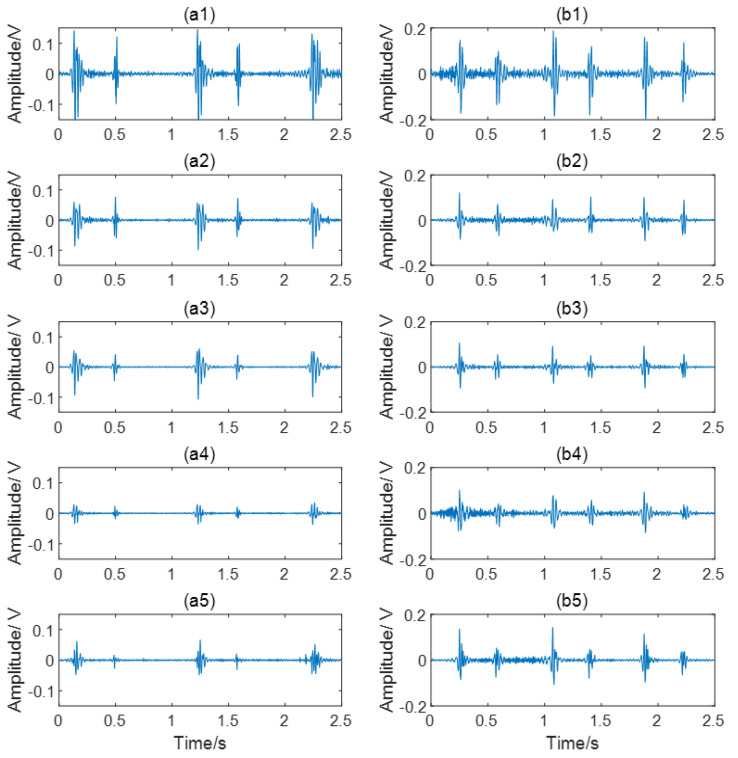
Collected heart sound signals. (**a1**–**a5**) The heart sound signals from channel 1 to channel 5 of a non-CAD subject; (**b1**–**b5**) The heart sound signals from channel 1 to channel 5 of a CAD patient.

In order to remove the interference of respiration, filtering is a necessary step in heart sound signal preprocessing. The advantage of the Butterworth filter is that the amplitude-frequency characteristic is flat and monotonous in the passband [28]. Although the attenuation of this filter in stopband is relatively slow, the fifth-order filter is still acceptable in this study. Therefore, a fifth-order Butterworth high pass filter with a cut-off frequency of 30 Hz was applied to remove the low-frequency noise and the baseline drift. Subsequently, a 50 Hz notch filter was used to remove power frequency interference. The comparison before and after preprocessing is shown in Figure 2. To enlarge the sample size, each five-minute recording was cropped to 30 segments lasting 10 s. Segments with wheezing of asthma or serious noise interference were considered unqualified. After quality evaluation and elimination [29], a total of 991 samples were generated, including 553 CAD and 438 non-CAD samples. The heart sounds from channel 1 to channel 5 of a non-CAD subject and a CAD patient are given in Figure 3. It can be observed that there are differences between the five channels, such as the amplitude ratio of first heart sound and second heart sound. There are also differences between the non-CAD subject and the CAD patient. The heart sound signals of the CAD group have more heart murmur.

### 2.3. Features Extraction

**Figure 4:**
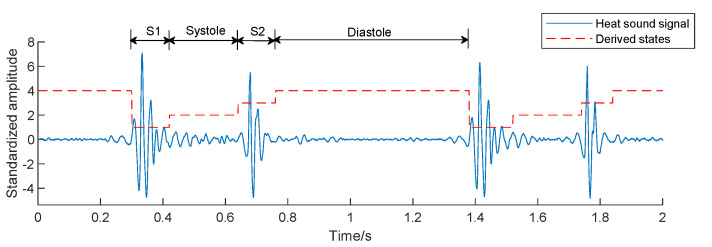
Heart sound signal after segmentation.

The segmentation of the fundamental heart sounds is an essential step in the automatic analysis of the heart sound signal. There are two main components in a cardiac cycle: The first heart sound (S1), caused by the closure of the mitral and tricuspid valves and their vibrations; the second heart sound (S2), generated by the closure of the aortic and pulmonary valves and their vibrations. The systole interval is the window between S1 and S2, and the diastole interval is from S2 to the beginning of S1 in the next heart cycle. For each cardiac cycle, the PCG signal was segmented into four states: S1, systole, S2, and diastole, using the algorithm proposed by Springer et al. [30]. The segmentation diagram is described in Figure 4. In this study, 20 time-domain, 16 frequency-domain, and 12 entropy features of each channel were extracted, and there were 240 single-channel features. 3 cross entropy features were extracted from every two channels. Since there were 10 combinations of five channels, 30 cross entropy features were extracted in this study.

#### 2.3.1. Time-Domain Features (20 × 5 Features)

The duration of each cardiac activity state often reflects changes in the state of the heart, since they are generated by the specific cardiac activities. The amplitude of heart sound can represent the intensity of cardiac mechanical activity, which may be potentially helpful for the detection of CAD. In this study, the mean value and standard deviation (SD) of interval durations, duration ratios, and average amplitude ratios were calculated [31]. The details are given in Table 2.

#### 2.3.2. Frequency-Domain Features (16 × 5 Features)

Frequency spectrum analysis is the most widely used approach in heart sound analysis. The fast Fourier transform (FFT) was used in this study. The normal heart sound signal generally had a frequency band below 200 Hz, and the noise related to diseases was generally ranging between 200 and 800 Hz [32]. At the same time, heart sound signals of CAD patients and non-CAD subjects were also significantly different at the low-frequency power of 25–60 Hz, especially at 31.5 Hz [9]. According to the existing research conclusions [33], 200 Hz and 50 Hz were used as the thresholds of high and low frequency. The spectrum ratios were extracted as frequency domain features, and a detailed description is presented in Table 3. First, the spectrum values of each state were calculated using fast Fourier transform. Then, the proportions of high frequency (above 200 Hz) and low frequency (below 50 Hz) components in the spectrum of four state spectra were obtained separately. Their mean values and SD were calculated as features. 

#### 2.3.3. Entropy Features (12 × 5 Features)

Entropy features were extracted including SampEn, FuzzyEn, and DistEn. For this work, the mean value and SD of entropy features in systole and diastole were calculated, and the details are given in Table 4.

1. SampEn is a nonlinear feature to calculate the probability of generating new patterns in signals [20]. It is also a common method to measure the complexity of time series [21]. SampEn can be calculated as follows:
  (1) $$SampEn\left(m,r,N\right)=-\ln\frac{\sum_{i=1}^{N-m}B_{i}^{\left(m+1\right)}\left(r\right)}{\sum_{i=1}^{N-m}B_{i}^{\left(m\right)}\left(r\right)},$$
  where *N* is the length of signals, *m* is the embedding dimension, *r* is the threshold parameter, and $B_{i}^{\left(m\right)}\left(r\right)$ is the probability that any two epochs match each other.
2. FuzzyEn [34] is actually a refined algorithm of SampEn. The difference between them lies in the thresholding procedure. The fuzzy membership function to determine the fuzzy similarity $S_{ij}^{m}$ between $X_{i}^{m}$ and $X_{j}^{m}$ is:
  (2) $$S_{ij}^{m}=\exp\left(-d_{ij}^{2}/r\right),$$
  where $d_{ij}$ is the distance between $X_{i}^{m}$ and $X_{j}^{m}$. In this study, for SampEn and FuzzyEn, the pattern length *m* was set to 2, and matching tolerance *r* was set to 0.2 times the SD of the input time series [35]. It has been shown in published studies that the introduction of the fuzzy membership function significantly improves the stability and consistency of the algorithm [36].
3. DistEn [23] uses empirical probability distribution functions (ePDF) to achieve the global measurement of the distance matrix, avoiding the parameter dependence caused by local evaluation. The ePDF of $d_{ij}^{m}$ is estimated using a histogram with a predefined bin number *B.* Then DistEn is defined by the Shannon formula for entropy:
  (3) $$DistEn(m)=-\frac{1}{\log_{2}(B)}\sum_{m=1}^{B}p_{t}\log_{2}(p_{t}),$$
  Thus, the range of DistEn should be within [0, 1]. In this study, B was set to 2^8.

#### 2.3.4. Cross Entropy Features (3 × 10 Features)

Coupling, also known as synchrony, was first proposed by Huygens [37]. XSampEn, XFuzzyEn, and JDistEn used in this study are accepted methods to measure coupling [38]. As the cross entropy features were extracted from every two channels, there were 10 combinations of five channels. Therefore, 30 cross entropy features were extracted in this study.

1. XSampEn [20] is developed from SampEn. It measures the synchronization of two signals by focusing on the similarity of patterns between two signals. XSampEn is defined as:
  (4) $$XSampEn\left(m,\tau ,r\right)=-\ln\frac{\sum_{i=1}^{N-m\tau }B_{i}^{\left(m+1\right)}\left(r\right)}{\sum_{i=1}^{N-m\tau }B_{i}^{\left(m\right)}\left(r\right)},$$
  where *m* is the embedding dimension, *τ* is the time delay and *r* is the threshold parameter.
2. XFuzzyEn [26] has the same algorithm framework as XSampEn. FuzzyEn substituted a Gaussian function for the Heaviside function as the membership function, i.e., the $B_{i}^{\left(m\right)}\left(r\right)$ is defined by:
  (5) $$B_{i}^{\left(m\right)}\left(r\right)=\frac{1}{N-m\tau }\sum_{j=1,j\neq i}^{N-m\tau }e^{-\ln(2){\left(\frac{d_{i,j}}{r}\right)}^{2}},$$
  The parameter *m* was set to 2 in this study. Since the signal is normalized, the SD is 1. In order to find out the best parameter *r*, the XSampEn and XFuzzyEn with *r* = 0.1, *r* = 0.15, *r* = 0.2, *r* = 0.25, and *r* = 0.3 were calculated. After comparing the results, *r* was set to 0.2.
3. The JDistEn algorithm [27] is developed by combining the joint distance matrix and DistEn. The ePDF of $d_{ij}^{m}$ is estimated by histogram with a predefined bin number *B*, which is denoted by $P_{t}$ where *t* = 1, 2, … *B*. Then JDistEn is defined by the Shannon formula for entropy:
  (6) $$JDistEn\left(m,\tau ,B\right)=-\frac{1}{\log_{2}\left(B\right)}\sum_{t=1}^{B}p_{t}\log_{2}\left(p_{t}\right),$$
  JDistEn has been shown to have especially good performance in short-length data [27]. In this study, the number of histogram bins *B* was set to 2^8.

### 2.4. Feature Set Construction

In order to explore whether the multi-channel signal features perform better and whether the two types of entropy features can improve the classification accuracy, five types of feature sets were established. Single-channel feature set 1 was composed of one-channel features without entropy features, which was abbreviated as ‘Sin–feature set 1’. Single-channel feature set 2 was composed of one-channel features with entropy features, which was abbreviated as ‘Sin–feature set 2’. Multi-channel feature set 1 included five-channel features without entropy features, which was abbreviated as ‘Mul–feature set 1’. Multi-channel feature set 2 included five-channel features with entropy features, which was abbreviated as ‘Mul–feature set 2’. Multi-channel feature set 3 included five-channel features with entropy features and cross entropy features, which was abbreviated as ‘Mul–feature set 3’. Sin–feature set 1 and Sin–feature set 2 represented five feature sets from channel 1 to channel 5, respectively.

### 2.5. Statistical Analysis

The generalized linear mixed model (GLMM) [39] is used for the statistical analysis in this paper. GLMM can be regarded as the fusion of the generalized linear model and linear mixed model, whose dependent variable need not satisfy the normal distribution. GLMM is suitable for processing repeated measurement data. The dependent variable of this study was the binary categorical variable, so the distribution of the fitted mixed model was set as binomial distribution, and the link function was set as a logit function. Statistical significance was set a priori at *p* < 0.05.

### 2.6. Feature Selection

Feature selection is particularly important. It is difficult to obtain a satisfactory performance by directly inputting the features into the classifier. This study used two feature selection methods including information gain and SVM–RFE to reduce feature dimension and enhance classification performance.

1. Information gain [40] is a statistic used to describe the ability to distinguish data samples. Features with larger information gain values are considered to contribute more to classification. Information gain is defined as information entropy minus conditional entropy.
2. SVM–RFE can repeatedly build SVM models to obtain the optimal feature subset. Features with the lowest contribution are iteratively eliminated from the training set, and the ranking from salient to non-salient features is generated [41]. Thus, the optimal feature subset is constructed by selecting the appropriate feature number.

### 2.7. Classification

The task of CAD classification is a typical binary classification problem. SVM was chosen in this study because of its excellent performance in small sample binary classification problems [42]. In n-dimensional space, SVM separates input data into the classes using hyperplanes. When the sample cannot be divided linearly, the kernel function is used to map the sample to a higher latitude space, and then find the hyperplane. The radial basis function kernel is a common kernel function of SVM, which contains two important hyper-parameters: C and gamma. The cost parameter C is used to control the overfitting of the model, and gamma is used to control the non-linear degree of the model [43]. According to previous experimental experience and relevant research [44], the detailed parameter configuration of the SVM classifier is shown in Table 5.

### 2.8. Performance Evaluation

Five-fold cross validation was performed in this work, and the final classification result was the average of five cross validations to make the evaluation more realistic. In order to ensure that the segments of the training group and the validation group came from completely different subjects, the recordings were divided into five parts firstly, and then every recording in each part was cropped into 30 segments lasting 10 s. Stratified sampling was used to ensure the balance of positive and negative samples.

In this study, the standard metrics including sensitivity (Se.), specificity (Sp.), and accuracy (Acc.) were used to measure the classification performance [45]. The equations associated with these metrics are calculated as

(7) $$\mathrm{Acc}.=\frac{TP+TN}{TP+TN+FP+FN},\;$$

(8) $$\mathrm{Se}.=\frac{TP}{TP+FN},$$

(9) $$\mathrm{Sp}.=\frac{TN}{TN+FP},$$

where *TP*, *TN*, *FP*, and *FN* stand for the number of the true positives, true negatives, false positives, and false negatives, respectively.

## 3. Results

In this study, the data pre-processing, feature extraction, and machine learning code were executed in Matlab R2019a. The entire experiment was implemented on a PC with a 3.70 GHz Intel Core i7-8700 k CPU, 16 GB of RAM, and a Windows 10 operating system.

### 3.1. Results Based on Statistical Analysis

In this paper, all the features were fitted by GLMM for statistical analysis. A total of 31 features were proven to be statistically different, including 5 time-domain features, 10 frequency-domain features, 2 entropy features, and 18 cross entropy features. The details of features with statistical differences are shown in Table 6. In the statistical analysis, 1 represented subject with CAD and 0 represented subject without CAD. Therefore, the odds ratio represented the increment of CAD odds for each 1 unit increased in the feature. In the comparison of the features of different domains, cross entropy features accounted for the largest proportion of the features with statistical differences, although the number of them was the least. The feature with the largest odds ratio was the frequency-domain feature. 

**Figure 5:**
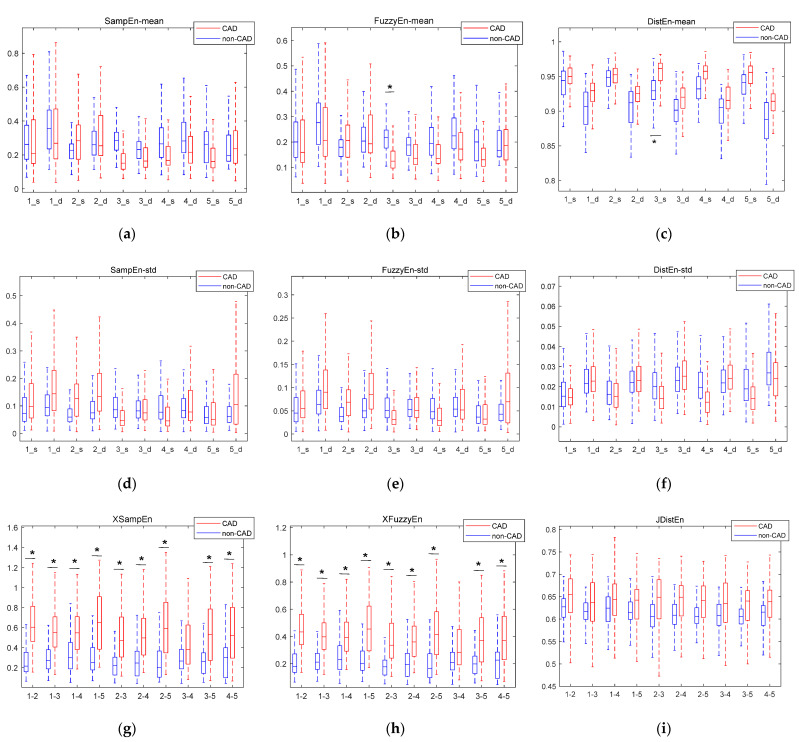
Boxplots of entropy features and cross entropy features. (**a**) The mean value of SampEn in different states; (**b**) The mean value of FuzzyEn in different states; (**c**) The mean value of DistEn in different states; (**d**) The standard deviation of SampEn in different states; (**e**) The standard deviation of FuzzyEn in different states; (**f**) The standard deviation of DistEn in different states; (**g**) XSampEn in every two channels; (**h**) XFuzzyEn in every two channels; (**i**) JDistEn in every two channels. Features marked with * are statistically significantly different.

The boxplots of entropy and cross entropy features are shown in Figure 5. The abscissa ‘1_s’ in (a)–(f) means systolic period of channel 1, and ‘1_d’ means diastolic period of channel 1. The abscissa ‘1–2’ in (g)–(h) represents the cross entropy feature extracted jointly by channel 1 and channel 2. Features marked with * are statistically significantly different. It can be seen that the XSampEn and XFuzzyEn on most channels had statistically significant differences between CAD patients and non-CAD subjects. The eigenvalues of XSampEn, XFuzzyEn, and JDistEn of CAD patients were generally larger than those of non-CAD subjects.

### 3.2. Ranking Results Based on Information Gain

**Figure 6:**
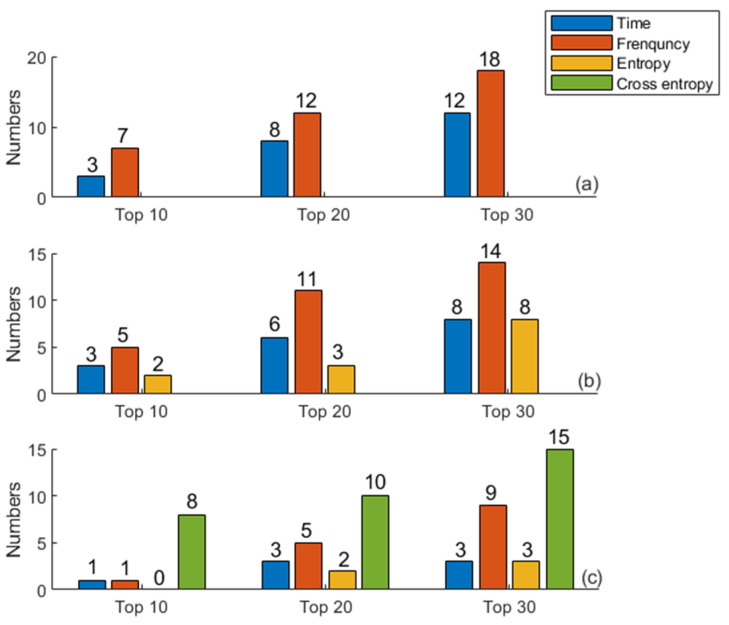
Numbers of different domain features in different feature sets. (**a**) Mul–feature set 1; (**b**) Mul–feature set 2; (**c**) Mul–feature set 3.

The value of information gain reflects the importance of features. In this study, information gain values of 270 features were calculated, and were sorted from large to small. In order to explore the importance of different domain features for CAD detection, the numbers of different domain features in the top 10, top 20, and top 30 are counted and shown in Figure 6. In Figure 6, Mul–feature set 1 was used in (a), Mul–feature set 2 was used in (b), and Mul–feature set 3 was used in (c). It can be seen that the cross entropy features and frequency domain features perform excellently in feature ranking, while entropy features perform mediocrely. As always, the performance of frequency domain features is better than that of the time domain feature.

### 3.3. Classification Performance

**Figure 7:**
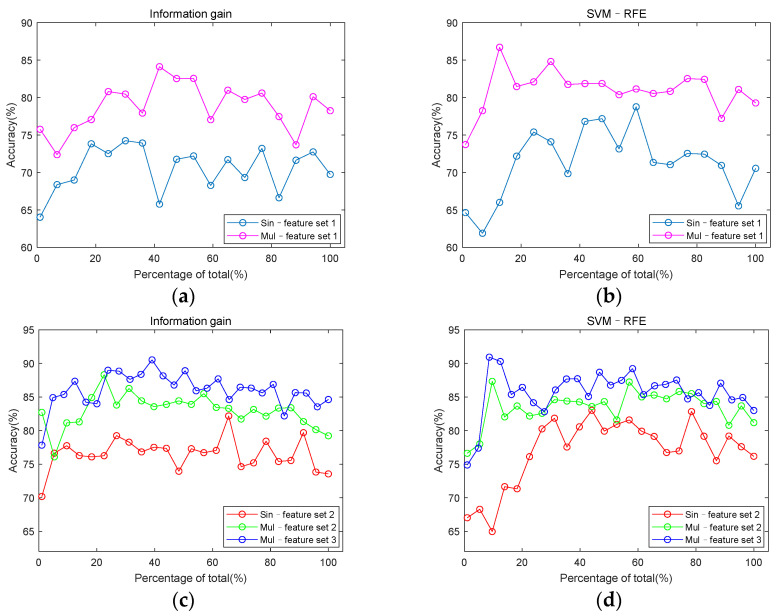
Comparison of classification accuracy between single-channel feature sets and multi-channel feature sets. (**a**) classification accuracy of Sin–feature set 1 and Mul–feature set 1 under information gain; (**b**) classification accuracy of Sin–feature set 1 and Mul–feature set 1 under SVM–RFE; (**c**) classification accuracy of Sin–feature set 2, Mul–feature set 2 and Mul–feature set 3 under information gain; (**d**) classification accuracy of Sin–feature set 2, Mul–feature set 2 and Mul–feature set 3 under SVM–RFE.

The information gain and SVM–RFE method were used to select features. After being sorted and selected, the features were put into the SVM classifier to compare whether the features from multi-channel signals performed better and whether entropy and cross entropy features can improve the classification accuracy. The number of features from single-channel feature sets was selected incrementing at step size 2, and the number of features from multi-channel feature sets was at step size 10. In order to explore the impact of multi-channel features on classification performance, classification accuracy based on single-channel feature sets and multi-channel feature sets were compared. The results are shown in Figure 7. It is worth noting that the number of features from different feature sets is different, so the abscissa of Figure 7 is a percentage of the total number of features. It can be clearly seen that multi-channel feature sets have advantages over single-channel feature sets in detecting CAD.

**Figure 8:**
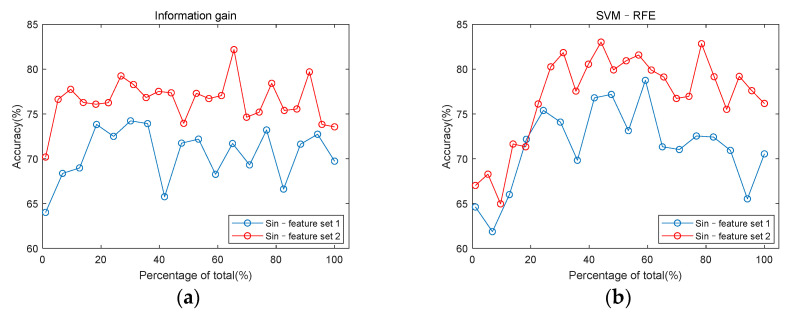

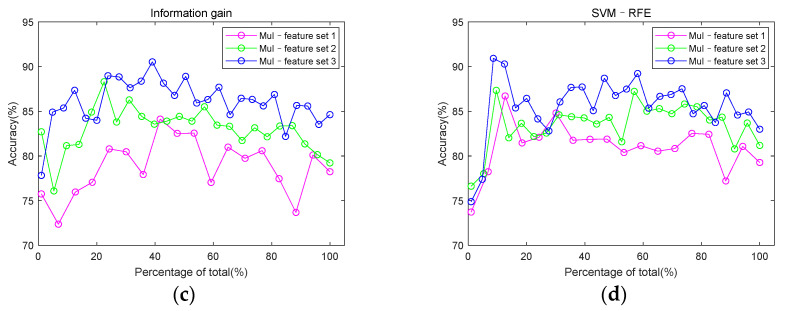
Comparison of classification accuracy with or without entropy features and cross entropy features. (**a**) classification accuracy of Sin–feature set 1 and Sin–feature set 2 under information gain; (**b**) classification accuracy of Sin–feature set 1 and Sin–feature set 2 under SVM–RFE; (**c**) classification accuracy of Mul–feature set 1, Mul–feature set 2 and Mul–feature set 3 under information gain; (**d**) classification accuracy of Mul–feature set 1, Mul–feature set 2, and Mul–feature set 3 under SVM–RFE.

Figure 8 uses the same data as Figure 7, it is drawn to explore the role of entropy and cross entropy features in classification. For the single-channel feature set, the highest classification accuracy among the five channels was used to draw Figure 7 and Figure 8. When using information gain, the feature set of channel 3 had the highest classification accuracy. When using SVM–RFE, the feature set of channel 2 had the highest classification accuracy. But the classification accuracy of the two channels differed by only 0.85%. It can be seen from Figure 8 that the accuracy of classification is improved by adding entropy and cross entropy features to either the single-channel feature set or the multi-channel feature set.

Table 7 and Table 8 shows the highest classification accuracy of each feature sets. After feature selection, the top 30 features of Mul–feature set 3 selected by SVM–RFE achieved the best performance with an accuracy of 90.92%. Besides, the top 22 features of Sin–feature set 2 selected by SVM–RFE achieved the best performance of single-channel features with an accuracy of 83.02 %.

## 4. Discussion

Considering the location of coronary artery occlusion, all CAD patients had left anterior descending stenosis, in which there were 7 subjects with first diagonal branch stenosis, 2 subjects with second diagonal branch stenosis, 1 subject with septal artery stenosis, 1 subject with middle branch stenosis and 16 subjects with left circumflex artery stenosis. That is to say, most coronary artery occlusion occurred in the left coronary artery. Among the five auscultation locations designed in this study, the positions of channel 2 and channel 3 mainly detected the left coronary artery. Therefore, channel 2 and channel 3 had excellent classification performance, which is consistent with our results. Previous studies [5,6] showed that coronary stenosis produces high-frequency sounds due to the turbulent blood flow in partially occluded arteries. This is consistent with the conclusion that the feature with the largest odds ratio in our study is the frequency domain feature.

Statistical analysis is an important step in exploring the validity of features. This study expands the sample size by segmenting the heart sound signal, so the samples of the same person are not independent of each other. Data segmentation is equivalent to the repeated measurement of data, which is suitable for analysis using GLMM. GLMM includes both fixed effects and random effects, and random effects can eliminate the influence of feature correlation within the group. The role of information gain is to estimate the importance of extracted features. The results show that the cross entropy feature not only accounts for the largest proportion in the top 10, top 20, and top 30, but also has the largest number of features that are statistically different. Considering that there are only 30 cross entropy features in the total 270 features, this result is more encouraging. SVM–RFE is a greedy algorithm for finding the optimal feature subset. Although it is time-consuming, it can enormously improve the accuracy of classification. The index of SVM–RFE is based on classification. Therefore, it is more reliable in improving the accuracy of classification.

Figure 7 shows that the classification performance using features extracted from multi-channel signals is better than that from single-channel signals. Compared with single-channel signals, multi-channel signals can provide more information about detecting CAD from suspected patients. The murmurs generated by coronary artery occlusion are more likely to be picked up by multiple heart sound sensors located at different locations. In other words, multi-channel signals acquisition can increase the probability of detecting CAD. In addition, in this study, the application of multi-domain features also plays a significant role. Previous studies have proven that features from multiple domains are more conducive to feature classification [33,46].

Figure 8 shows the comparison of classification accuracy before and after adding the entropy feature. It can be seen that the accuracy of classification is improved by adding entropy and cross entropy features. Figure 5g–i show that the cross entropy features of CAD patients have a consistent increase compared with non-CAD. Cross entropy is a physical quantity to represent synchronization. The increase of the cross entropy represents a decrease in the synchronization of the two signals. For CAD patients, the stenosis of blood vessels will lead to myocardial ischemia and reduction of myocardial contractility. In cases of myocardial ischemia, the energy supply decreases, which can result in systolic dysfunction, such as delayed contraction, decreased contraction force, and non-synchronized motion in the myocardium. Changes in the state of the heart affect the flow of blood, which is reflected in the heart sound and captured by a microphone on the body surface. Previous studies concluded that there is disturbed myocardial synchrony in CAD patients, with greater dyssynchrony than in the control group [47]. This is consistent with the increase of the cross entropy of CAD patients in Figure 5.

Table 9 summarizes the existing studies that use heart sound signals for CAD detection. Among these studies, the highest accuracy of CAD detection using heart sound signals is 84% [17]. Most of the studies in Table 9 identified CAD patients and healthy subjects. Obviously, it is easier to identify CAD patients and healthy subjects, because of their obvious differences in clinical symptoms and examination results. However, for the similarity of clinical symptoms, metabolism and electrocardiogram between CAD and suspected CAD patients [48], it is very difficult for physicians to accurately diagnose them. A previous study concluded that 10–30% of patients who received CAG due to angina pectoris had “normal” or “near normal” coronary arteries during CAG [49], which causes an additional significant burden on patients, families, and society. In this study, CAD and suspected CAD patients were identified, and the classification accuracy of the multi-domain features extracted from multi-channel signals is 90.92%.

## 5. Conclusions

Among all the single-channel features, the highest classification accuracy was 83.02%. After collecting heart sound signals from five different locations, the classification accuracy of the multi-channel features was 86.70%. After adding entropy features and cross entropy features, the classification accuracy improved to 90.90%. It is concluded that multi-channel heart sounds can provide further information for CAD detection, and entropy features and cross entropy features have the advantage of improving classification accuracy. Due to the advantages of non-invasive, low cost, and simple operation, the use of heart sound signals will inevitably provide great help in disease screening and detection. Cross entropy features have shown great potential in statistical analysis and feature ranking, and more in-depth research can be done in the future. Multi-domain feature fusion of multi-channel heart sound signals can provide additional information, which will play an important role in the process of preventing and overcoming cardiovascular diseases. In future work, signals from more subjects are necessary to test the performance of the proposed method further. Moreover, we will pay attention to exploring more useful features and classification methods.

## Figures and Tables

**Table 1:** Basic characteristics of all subjects.

Characteristic	CAD	Non-CAD	*p* Value
Age (year)	57 ± 9	54 ± 7	0.27
Male/Female	12/9	9/6	0.57
Height (cm)	166 ± 7	167 ± 7	0.61
Weight (kg)	73 ± 10	74 ± 7	0.59
Body mass index (kg/m^2^)	27 ± 3	26 ± 2	0.90
Systolic blood pressure (mmHg)	135 ± 16	137 ± 11	0.48
Diastolic blood pressure (mmHg)	81 ± 15	80 ± 13	0.95
Heart rate (beats/min)	73 ± 13	79 ± 12	0.21

Note: values are expressed as male/female or mean value ± standard deviation.

**Table 2:** Extracted time-domain features during a cardiac cycle of each channel.

Abbreviation	Description
CC	The cardiac cycle duration
IntS1	The S1 interval duration
IntS2	The S2 interval duration
IntSys	The systolic interval duration
IntDia	The diastolic interval duration
Ratio_SysCC	The ratio of systolic interval to the cardiac cycle duration
Ratio_DiaCC	The ratio of diastolic interval to the cardiac cycle duration
Ratio_SysDia	The ratio of systole interval to the diastole interval
Ratio_Amp_SysS1	The ratio of average amplitude during systole to that during S1
Ratio_Amp_DiaS2	The ratio of average amplitude during diastole to that during S2

**Table 3:** Extracted frequency-domain features during a cardiac cycle of each channel.

Abbreviation	Description
HFAll_S1	The proportion of high-frequency component in total spectrum S1s
LFAll_S1	The proportion of low-frequency component in total spectrum S1s
HFAll_S2	The proportion of high-frequency component in total spectrum S2s
LFAll_S2	The proportion of low-frequency component in total spectrum S2s
HFAll_Sys	The proportion of high-frequency component in total spectrum systoles
LFAll_Sys	The proportion of low-frequency component in total spectrum systoles
HFAll_Dia	The proportion of high-frequency component in total spectrum diastoles
LFAll_Dia	The proportion of low-frequency component in total spectrum diastoles

**Table 4:** Extracted entropy features during a cardiac cycle of each channel.

Abbreviation	Description
SampEn_Sys	The sample entropy of systolic
SampEn_Dia	The sample entropy of diastolic
FuzzyEn_Sys	The fuzzy entropy of systolic
FuzzyEn_Dia	The fuzzy entropy of diastolic
DistEn_Sys	The distribution entropy of systolic
DistEn_Dia	The distribution entropy of diastolic

**Table 5:** Detailed parameter configuration of the SVM classifier.

Parameter	Instructions
C	‘2^−5^–2^5^’
Gamma	‘2^−5^–2^5^’
Kernel function	‘radial basis function’
Scoring	‘accuracy’
Cv	5
Class_weight	‘balanced’

**Table 6:** Details of features with statistical differences.

Feature	Domain	Odds Ratio	*p* Value	Feature	Domain	Odds Ratio	*p* Value
XSampEn_12	Cro-en	3.86 × 10^7^	0.00	m_Amp_SysS1_1	Time	1.17	0.01
XSampEn_13	Cro-en	5.01 × 10^7^	0.00	m_LFAll_Sys_1	Frequency	9.63 × 10^−25^	0.01
XSampEn_14	Cro-en	2.74 × 10^4^	0.01	m_LFAll_Dia_1	Frequency	1.56 × 10^−22^	0.01
XSampEn_15	Cro-en	6.29 × 10^4^	0.00	m_Amp_SysS1_2	Time	1.25	0.00
XSampEn_23	Cro-en	1.24 × 10^6^	0.00	m_HFAll_S1_2	Frequency	3.23 × 10^61^	0.00
XSampEn_24	Cro-en	1.02 × 10^4^	0.01	m_LFAll_S1_2	Frequency	1.45 × 10^−21^	0.01
XSampEn_25	Cro-en	4.53 × 10^4^	0.00	m_LFAll_Sys_2	Frequency	1.56 × 10^−18^	0.03
XSampEn_35	Cro-en	7.19 × 10^3^	0.01	m_LFAll_S2_2	Frequency	2.91 ×10^−22^	0.00
XSampEn_45	Cro-en	4.33 × 10^2^	0.04	m_LFAll_Dia_2	Frequency	3.34 × 10^−22^	0.01
XFuzzyEn_12	Cro-en	3.39 × 10^11^	0.00	m_Amp_SysS1_3	Time	1.35	0.00
XFuzzyEn_13	Cro-en	9.99 × 10^11^	0.00	m_FuzzyEn_Sys_3	Entropy	6.40 × 10^−10^	0.03
XFuzzyEn_14	Cro-en	5.78 × 10^6^	0.01	m_DistEn_Sys_3	Entropy	5.33 × 10^30^	0.02
XFuzzyEn_15	Cro-en	2.30 × 10^7^	0.00	m_Amp_SysS1_4	Time	1.21	0.03
XFuzzyEn_23	Cro-en	2.15 × 10^9^	0.00	m_Amp_SysS1_5	Time	1.26	0.02
XFuzzyEn_24	Cro-en	1.07 × 10^6^	0.01	m_HFAll_S1_5	Frequency	2.14 × 10^26^	0.03
XFuzzyEn_25	Cro-en	1.00 × 10^7^	0.00	m_LFAll_Sys_5	Frequency	5.69 × 10^−15^	0.04
XFuzzyEn_35	Cro-en	8.56 × 10^5^	0.01	m_LFAll_Dia_5	Frequency	1.22 × 10^−15^	0.03
XFuzzyEn_45	Cro-en	8.18 × 10^3^	0.04				

Note: ‘Cro-en’ is short for ‘Cross entropy’, the odds ratio is represented by scientific notation, and the suffix number indicates the channel.

**Table 7:** Comparison of the best classification performance of different single-channel feature sets under different selection methods.

	Information Gain						SVM–RFE					
	Without Entropy			With Entropy			Without Entropy			With Entropy		
	Acc. (%)	Se. (%)	Sp. (%)	Acc. (%)	Se. (%)	Sp. (%)	Acc. (%)	Se. (%)	Sp. (%)	Acc. (%)	Se. (%)	Sp. (%)
Ch-1	75.94 ± 8.19	73.38 ± 15.41	77.56 ± 11.07	80.57 ± 5.98	79.87 ± 21.92	81.23 ± 17.22	77.55 ± 11.59	74.45 ± 23.12	80.14 ± 14.01	79.41 ± 7.02	77.75 ± 18.19	81.78 ± 10.62
Ch-2	77.92 ± 10.52	73.00 ± 13.36	81.71 ± 10.72	80.77 ± 8.50	73.65 ± 24.95	87.38 ± 12.84	78.75 ± 6.94	79.93 ± 18.75	78.32 ± 4.43	**83.02 ± 11.99**	80.39 ± 18.72	84.07 ± 18.35
Ch-3	74.09 ± 8.35	73.03 ± 15.08	74.11 ± 9.96	**82.17 ± 6.55**	78.89 ± 17.52	85.31 ± 11.18	74.52 ± 10.99	70.77 ± 26.94	78.55 ± 16.88	82.38 ± 8.18	76.42 ± 8.97	87.24 ± 9.75
Ch-4	67.86 ± 9.37	66.28 ± 20.86	71.27 ± 25.60	69.03 ± 15.79	69.52 ± 14.52	69.48 ± 20.05	61.86 ± 10.99	50.79 ± 17.94	71.24 ± 14.16	66.76 ± 5.43	63.12 ± 17.49	68.76 ± 16.72
Ch-5	68.48 ± 11.94	66.63 ± 6.98	70.81 ± 20.60	70.33 ± 5.80	68.97 ± 18.48	70.88 ± 5.57	70.10 ± 9.13	66.51 ± 16.92	72.80 ± 19.74	79.69 ± 12.78	75.79 ± 20.13	83.03 ± 14.60

Note: the bold format represents the highest classification accuracy in each selecting method, ‘Ch-1’ means ‘Channel 1’, and data are expressed as mean value ± standard deviation.

**Table 8:** Comparison of the best classification performance of different multi-channel feature sets under different selection methods.

	Information Gain			SVM–RFE		
	Acc. (%)	Se. (%)	Sp. (%)	Acc. (%)	Se. (%)	Sp. (%)
Mul–feature set 1	84.11 ± 5.47	75.76 ± 14.26	90.98 ± 6.42	**86.70 ± 6.42**	80.89 ± 16.74	91.01 ± 11.85
Mul–feature set 2	**88.30 ± 7.27**	79.09 ± 13.07	95.06 ± 6.70	87.33 ± 8.55	80.28 ± 15.77	92.90 ± 3.50
Mul–feature set 3	90.52 ± 5.67	80.66 ± 14.81	98.30 ± 2.85	**90.92 ± 6.89**	87.96 ± 8.71	93.04 ± 9.30

Note: the bold format represents the highest classification accuracy in each selecting method, and data are expressed as mean value ± standard deviation.

**Table 9:** Summary of the existing studies on the detection of CAD using PCG signals.

Author	Database	Feature & Classifier	Result (%)
Gauthier et al. [10] (2007)	30 subjects: 24 CAD & 6 normal	Fast Fourier Transform Optimal threshold detection	Acc. = 73.3 Se. = 71.0 Sp. = 83.0
Akay et al. [15] (2009)	40 subjects: 30 CAD & 10 normal	Approximate entropy Optimal threshold detection	Acc. = 77.0 Se. = 78.0 Sp. = 80.0
Griffel et al. [14] (2012)	31 subjects: 16 CAD & 15 non-CAD	Automutual information function Linear support vector machine classifier	Acc. = 81.0 Se. = 87.0 Sp. = 85.0
Schmidt et al. [9] (2015)	133 subjects: 63 CAD & 70 non-CAD	Frequency and nonlinear features Quadratic discriminant function	Acc. = 68.5 Se. = 72.0 Sp. = 65.2
Akanksha et al. [17] (2017)	50 subjects: 25 CAD & 25 normal	Cross power spectral density Support vector machine classifier	Acc. = 84.0 Se. = 82.0 Sp. = 81.3
Pathak et al. [19] (2020)	80 subjects: 40 CAD & 40 normal	Imaginary part of cross power spectral density Support vector machine classifier	Acc. = 75.0 Se. = 76.5 Sp. = 73.5
This paper	36 subjects: 21 CAD & 15 non-CAD	Multi-domain and multi-channel features Support vector machine classifier	Acc. = 90.9 Se. = 88.0 Sp. = 93.0

## Data Availability

The data presented in this study are available on reasonable request from the corresponding author, X.W. The data are not publicly available due to their containing information that could compromise the privacy of research participants.
